# Genotype–phenotype analysis and functional study of three novel *LRP6* variants in non-syndromic oligodontia

**DOI:** 10.3389/fgene.2025.1598907

**Published:** 2025-06-04

**Authors:** Yunyun Yuan, Ya Zhao, Lingqiang Meng, Shuyun Zheng, Hui Li, Jiabao Ren, Beibei Li, Chenyun Dou, Yan Hou, Wenjing Chen, Jing Zhang, Yulin Ding, Wenjing Shen

**Affiliations:** ^1^ Department of Prosthodontics, Hebei Key Laboratory of Stomatology, Hebei Technology Innovation Center of Oral Health, School and Hospital of Stomatology, Hebei Medical University, Shijiazhuang, China; ^2^ Department of Orthodntics, Hebei Key Laboratory of Stomatology, Hebei Technology Innovation Center of Oral Health, School and Hospital of Stomatology, Hebei Medical University, Shijiazhuang, China; ^3^ Department of Prosthodontics, Hebei Eye Hospital, Xingtai, China; ^4^ Department of Stomatology, The No. 2 Hospital of Baoding, Baoding, China; ^5^ Hebei Key Laboratory of Stomatology, Hebei Technology Innovation Center of Oral Health, School and Hospital of Stomatology, Hebei Medical University, Shijiazhuang, China

**Keywords:** tooth agenesis, non-syndromic oligodontia, LRP6, β-catenin, genotype-phenotype

## Abstract

**Introduction:**

Tooth agenesis (TA) is a common craniofacial malformation in humans, characterized by the absence of one or more permanent teeth. Recent studies have identified the low-density lipoprotein receptor-related protein 6 (*LRP6*) gene as an autosomal dominant contributor to TA. Herein we aimed to identify novel *LRP6* variants in patients with non-syndromic oligodontia (NSO) and perform functional analyses of these variants.

**Methods:**

Whole-exome sequencing (WES) was conducted on probands and their first-degree relatives to identify potential pathogenic variants. Identified LRP6 variants underwent computational pathogenicity prediction using integrated bioinformatics tools. Subcellular localization patterns were analyzed via immunofluorescence microscopy. Functional characterization of WNT/β-catenin signaling alterations was achieved through Western blot analysis and dual-luciferase reporter assays (TOP-Flash/FOP-Flash systems). Finally, genotype-phenotype correlations in *LRP6*-associated non-syndromic oligodontia (NSO) were systematically investigated.

**Results:**

We identified three novel *LRP6* variations (NM_002336): a truncating variant [c.2182C>T (p.Arg728*)] and two missense variants [c.3773C>T (p.Thr1258Met) and c.1441C>T (p.Arg481Cys)]. Immunofluorescence characterization revealed that the missense variants exhibited subcellular localization patterns comparable to wild-type LRP6, with predominant distribution in the plasma membrane and cytoplasmic compartments. Western blot analysis revealed impaired β-catenin expression in cells harboring the *LRP6* missense variants, suggesting compromised canonical WNT signaling pathway activity. Functional assessment using the TOP/FOP-Flash luciferase reporter system demonstrated significantly reduced *TCF/LEF* transcriptional activity associated with these variants, though statistical significance was exclusively observed for the Arg481Cys variant (*P* < 0.05). Literature review identified 39 *LRP6* variants associated with 52 NSO patients, revealing that mandibular second premolars were the most frequently affected teeth, while maxillary first molars were least likely to be affected.

**Discussion:**

We identified three novel *LRP6* variants in patients with NSO from three Chinese families. Furthermore, we have confirmed through *in vitro* experiments that these novel *LRP6* missense variants lead to impaired activation of the WNT signalling pathway. Finally, we summarized the genotype–phenotype correlation for *LRP6*-related NSO, finding that *LRP6* variants are most likely to affect the mandibular second premolars.

## 1 Introduction

Different degrees of congenital tooth loss, morphological abnormalities, and aberrant eruption are collectively referred to as tooth agenesis, a condition that affects permanent dentition more frequently than any other hereditary developmental disease encountered in dental clinics. The incidence of congenital tooth loss (excluding the third molar) ranges from 2.7% to 12.2% (Kiliaridis et al., 2016; Rakhshan, 2015). Non-syndromic oligodontia (NSO) refers to congenital tooth loss affecting at least six permanent teeth (excluding the third molar) without the involvement of other organ or system abnormalities. The prevalence of NSO varies by ethnicity: 0.1% in Finnish (Nieminen, 2009), and 0.13% in Turkish populations (Celikoglu et al., 2010). In the Caucasian population, its prevalence is estimated to be 0.14% (Créton et al., 2007), while in China, the prevalence among the Asian population is reported to be 0.25% (Feng, 2011).

Tooth development involves a complex interaction between epithelial and mesenchymal cells, regulated by numerous transcription factors, morphogenetic factors, and signaling pathways (Balic and Thesleff, 2015). Any early interference or injury to this process can lead to the failure of tooth development (Juuri and Balic, 2017). Both environmental and genetic factors contribute to the etiology of tooth agenesis (Fauzi et al., 2018). Environmental influences include trauma, chemotherapy, radiation, and thalidomide use during pregnancy. Genetically, tooth agenesis can follow X-linked, autosomal dominant, and autosomal recessive inheritance patterns (Yang et al., 2020), with gene variants accounting for most instances.

According to (Yu et al., 2019), variants in six genes—*PAX9* (paired box gene 9), *AXIN2* (axis inhibition protein 2), *EDA* (ectodysplasin A), *LRP6* (low-density lipoprotein receptor-related protein 6), *MSX1* (muscle segment homeobox 1), *WNT10A* (wingless-type MMTV integration site family, member 10A)—are responsible for approximately 91.9% of non-syndromic congenital tooth loss. LRP6, AXIN2, and WNT10A play key roles in the WNT signaling pathway, one of the primary pathways regulating tooth development (Duan and Bonewald, 2016; Hermans et al., 2021). LRP6 is a receptor in the WNT signaling pathway, forming a complex with WNT and Frizzled proteins to activate this pathway (Ockeloen et al., 2016; Tamai et al., 2004).

In humans, the *LRP6*, located on chromosome 12p13.2, spans 150 kb and consists of 23 exons; it encodes a protein of 1,613 amino acids (Wang et al., 2018). LRP6 includes an extracellular domain, a transmembrane domain, and a short intracellular domain. The extracellular domain is further divided into three segments: YWTD-β-propeller-EGF domains 1 and 2 (P1E1-P2E2), YWTD-propeller domains-EGFs YWTD-β-propeller-EGF domains 3 and 4 (P3E3-P4E4), and three LDLR a-type repeat sequences. The intracellular domain contains a PPPSP (Pro-Pro-Pro-Ser-Pro) motif (Wang et al., 2018). Variants in *LRP6* have been associated with syndromic tooth agenesis, often accompanied by cleft lip and/or palate and minor congenital abnormalities of the fingers and ears (Ockeloen et al., 2016; Basha et al., 2018; Dinckan et al., 2018; Ross et al., 2019). In 2015, Massink et al. identified *LRP6* as a novel candidate gene for NSO, underscoring the importance of the WNT signaling pathway in the etiology of human tooth agenesis.

Herein we screened 35 NSO families and identified one unique *LRP6* truncated variant (c.2182C>T, p. Arg728*) and two novel missense variants (c.3773C>T, p. Thr1258Met and c.1441C>T, p. Arg481Cys). Bioinformatics analysis was performed to establish a theoretical framework for genetic counseling and to expand the spectrum of non-syndromic congenital tooth loss. *In vitro* functional analysis revealed that the activation of WNT/β-catenin signaling was severely impaired by these *LRP6* variants. We further summarized the genotypes and phenotypes of these variants, providing a theoretical basis for inferring genotypes from clinical phenotypes.

## 2 Methods and materials

### 2.1 Study subjects

Thirty-five unrelated individuals with NSO were recruited for this study through referrals from the Department of Prosthodontics at Hebei Medical University Hospital of Stomatology, China, between 2017 and 2022. None of the patients had lost permanent teeth due to extractions or trauma. Each patient underwent a thorough intraoral examination and panoramic radiography to confirm the number (≥6) and distribution of missing teeth. There were no developmental abnormalities in other organs. One hundred healthy participants served as controls. The study was approved by the Ethics Committee of the School and Hospital of Stomatology, Hebei Medical University (no [2016] 004), and all participants provided written informed consent.

### 2.2 Peripheral blood collection and DNA extraction

Peripheral venous blood samples (2 mL/person) were collected from probands, available family members, and healthy participants. Genomic DNA was subsequently extracted from these samples using a kit (Beijing Tiangen Biochemical Technology Co., Ltd.), as per manufacturer instructions, and stored at −20°C for later use.

### 2.3 Whole-exome sequencing, sanger sequencing, and pathogenicity prediction

Genomic DNA from core family members was sent to iGeneTech for whole-exome sequencing (Beijing, China). The DNA library was prepared, and target exons were sequenced on the Nova6000 platform (Illumina Inc., United States). Variants were selected based on the following criteria: minor allele frequency <0.01 in ExAC or 1,000 Genome Databases; known pathogenic genes (e.g., *PAX9*, *AXIN2*, *EDA*, *LRP6*, *MSX1*, *WNT10A*, and *WNT10B*); and genes predicted to be pathogenic by Sorting Intolerant from Tolerant (SIFT) (Sim et al., 2012), PolyPhen-2 (Adzhubei et al., 2010), and Mutation Taster (Schwarz et al., 2014). Sanger sequencing was performed to verify *LRP6* variants in probands and healthy controls. NM_002336 was used as the reference sequence for *LRP6*.

### 2.4 Conservation and structural modeling of LRP6 variants

Conservation analysis of LRP6 amino acid sequences was performed across six species—human (NP_002327.2), cattle (>XP_002687829.2), dog (>XP_038294916.1), house mouse (>NP_032540.2), rhesus monkey (>NP_001244648.1), and pig (>XP_020948003.1)—using sequences obtained from the UniProtKB database (https://www.ncbi.nlm.nih.gov/). Multiple sequence alignment was conducted using Clustal Omega (https://www.ebi.ac.uk/Tools/msa/clustalo/), and sequence logos were generated using WebLogo v2.8.2 (http://weblogo.berkeley.edu/).

For tertiary structural analysis, LRP6 structure was retrieved from the Protein Data Bank (http://www.rcsb.org/). Structural changes were examined and visualized using PyMOL v2.1 (Molecular Graphics System, DeLano Scientific, CA, United States).

### 2.5 Construction of *LRP6* expression vectors

Full-length *LRP6* cDNA was subcloned into pcDNA3.1 (Honor Gene, Shanghai, China) with a C-terminal 3XFlag tag. Site-directed mutagenesis was used to generate the two missense variants of *LRP6* (c.3773C>T and c.1441C>T), and these were verified by Sanger sequencing (Honor Gene, Shanghai, China).

### 2.6 Cell culture, transient transfection, and immunofluorescence assay

Human embryonic kidney 293T (HEK-293T) cells were cultured in Dulbecco’s modified Eagle’s medium (Gibco, United States) supplemented with 10% fetal bovine serum (Gibco) at 37°C in a humidified atmosphere. Transient transfections were performed using Lipofectamine 3,000 (Thermo Fisher Scientific, MA, United States). Forty-eight hours post-transfection, samples were incubated with an anti-FLAG mouse-derived primary antibody (Sigma, F1804, United States) and then with a fluorescently labeled secondary antibody (Abways, AB0152, China) in the dark. Cells were stained with 4′,6-diamidino-2-phenylindole (Beyotime biotechnology, C1006, China) and then mounted. Images were acquired using an Olympus FV1000 laser confocal microscope (Olympus, Japan) (Zhao et al., 2024).

### 2.7 TOP-Flash/FOP-Flash luciferase reporter assay

Luciferase assay was detected according to the experimental methods of previous research of our team (Shen et al., 2016; Wang et al., 2020). An equal amount of 0.5 µg of *LRP6* plasmids (including the empty vector, wild type, p. Thr1258Met, and p. Arg481Cys) was co-transfected with 0.1 µg of the TOP-Flash/FOP-Flash reporter plasmid and 0.01 µg of the Renilla reporter plasmid (GeneChem, Shanghai, China) into HEK-293T cells, respectively. Renilla luciferase activity reporter served as an internal reference. Forty-eight hours post-transfection, cell lysates were analyzed firefly and Renilla luciferase activity in replicates using the dual-luciferase reporter assay system (Promega, E1910, United States). Firefly luciferase activity was normalized to Renilla luciferase for each sample. Each experiment was performed in triplicate and repeated at least 5 times. Statistical differences in relative luciferase activity between the wild-type and mutant samples were assessed using one-way ANOVA. Data were presented as mean ± SD (n = 3) (Yu et al., 2021).

### 2.8 Western blotting

HEK-293T cells transfected with the empty vector and the wild-type and mutant *LRP6* plasmids using Lipofectamine 3,000 (Thermo Fisher Scientific, MA, United States) were lysed in a lysis buffer (Beijing Solarbio Science & Technology Co., Ltd). Lysates were normalized, subjected to sodium dodecyl-sulfate polyacrylamide gel electrophoresis, and transferred to polyvinylidene difluoride membranes (Merck Millipore, DEU). Membranes were probed with anti-β-catenin (Abways, CY3523, China) and anti-β-actin primary antibodies (Abways, AB0011, China). After blocking with 5% milk in Tris-buffered saline and Tween 20 [50 mM Tris-HCl (pH 7.5), 150 mM NaCl, 0.1% Tween 20], membranes were incubated with appropriate secondary antibodies (Abbkine, A23920, China). Protein band intensities were quantified with ImageJ, with β-catenin band densities normalized to β-actin. One-way ANOVA was used to analyze phase statistical differences between groups (Song et al., 2023).

### 2.9 Genotype–phenotype analysis

A literature review was conducted using PubMed up to 30 June 2024, with the search terms “LRP6 variants” or “LRP6 mutations.” Reports lacking detailed phenotype information were excluded. Data from 51 *LRP6* variants, including those from three patients in this study, were used for genotype–phenotype analysis. Missing teeth numbers and rates were estimated.

## 3 Results

### 3.1 Clinical findings

**FIGURE 1:**
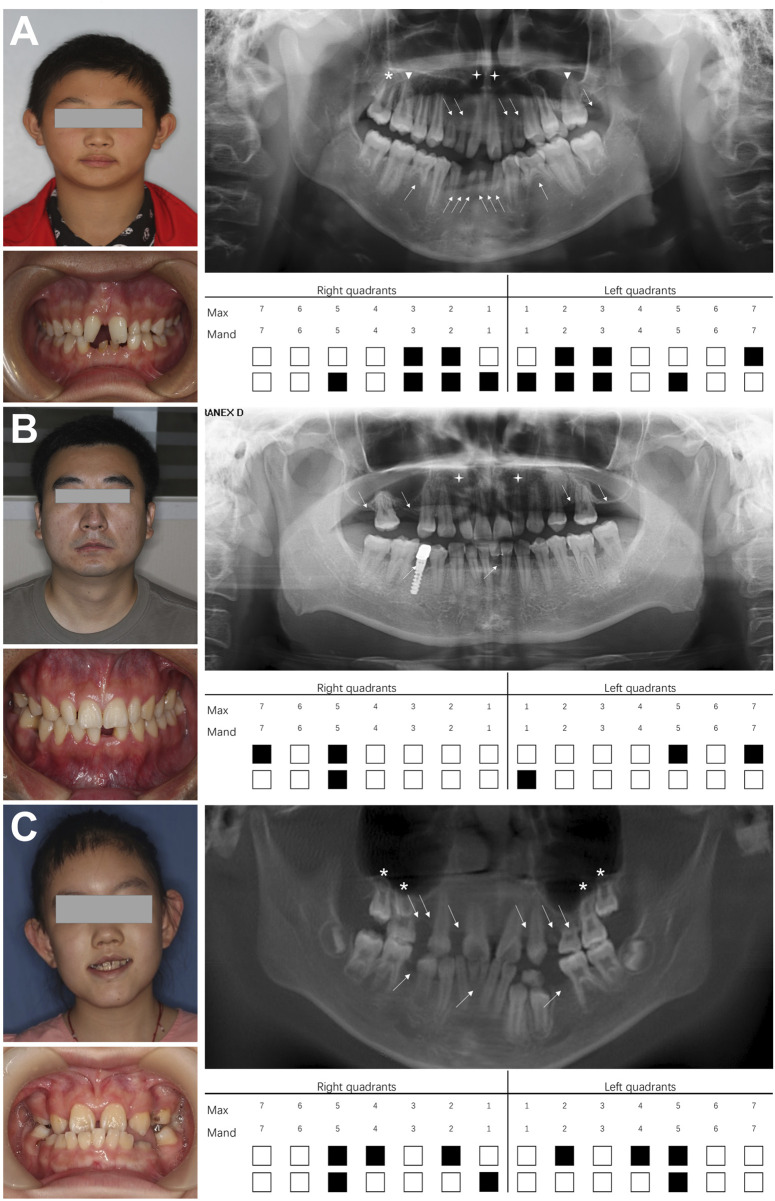
Dental characteristics of three probands with non-syndromic oligodontia. **(A)** Facial photographs, intraoral images, panoramic radiographs, and schematic of missing teeth for proband 1, **(B)** proband 2, and **(C)** proband 3. White arrows indicate missing teeth; white triangles represent unseparated tooth roots; asterisks indicate taurodontism; crosses indicate microdontia. Max, maxillary; Mand, mandibular.

All three NSO probands exhibited at least six missing teeth (excluding third molars) without abnormal development of skin, hair, or other organs. Proband 1, a 12-year-old male, had 13 missing teeth (12, 13, 22, 23, 27, 31, 32, 33, 35, 41, 42, 43, 45) (Figure 1A). Panoramic radiography revealed microdontia of the upper central incisor, long and unseparated roots in the upper first molars, and taurodontism in the upper second molars. His mother had a normal phenotype, while his father was missing teeth. Proband 2, a 27-year-old male, had six missing teeth (15, 17, 25, 27, 31, 45) and two conical teeth (12 and 22) (Figure 1B). His father had a normal phenotype, while his mother had congenital tooth loss. Proband 3, an 8-year-old girl, had nine missing teeth (12, 14, 15, 22, 24, 25, 35, 41, 45) (Figure 1C). X-rays showed taurodontism in all maxillary molars. Her father also had congenital tooth loss, while her mother had a normal phenotype.

### 3.2 Identification of novel *LRP6* variants altering LRP6 structure

**FIGURE 2:**
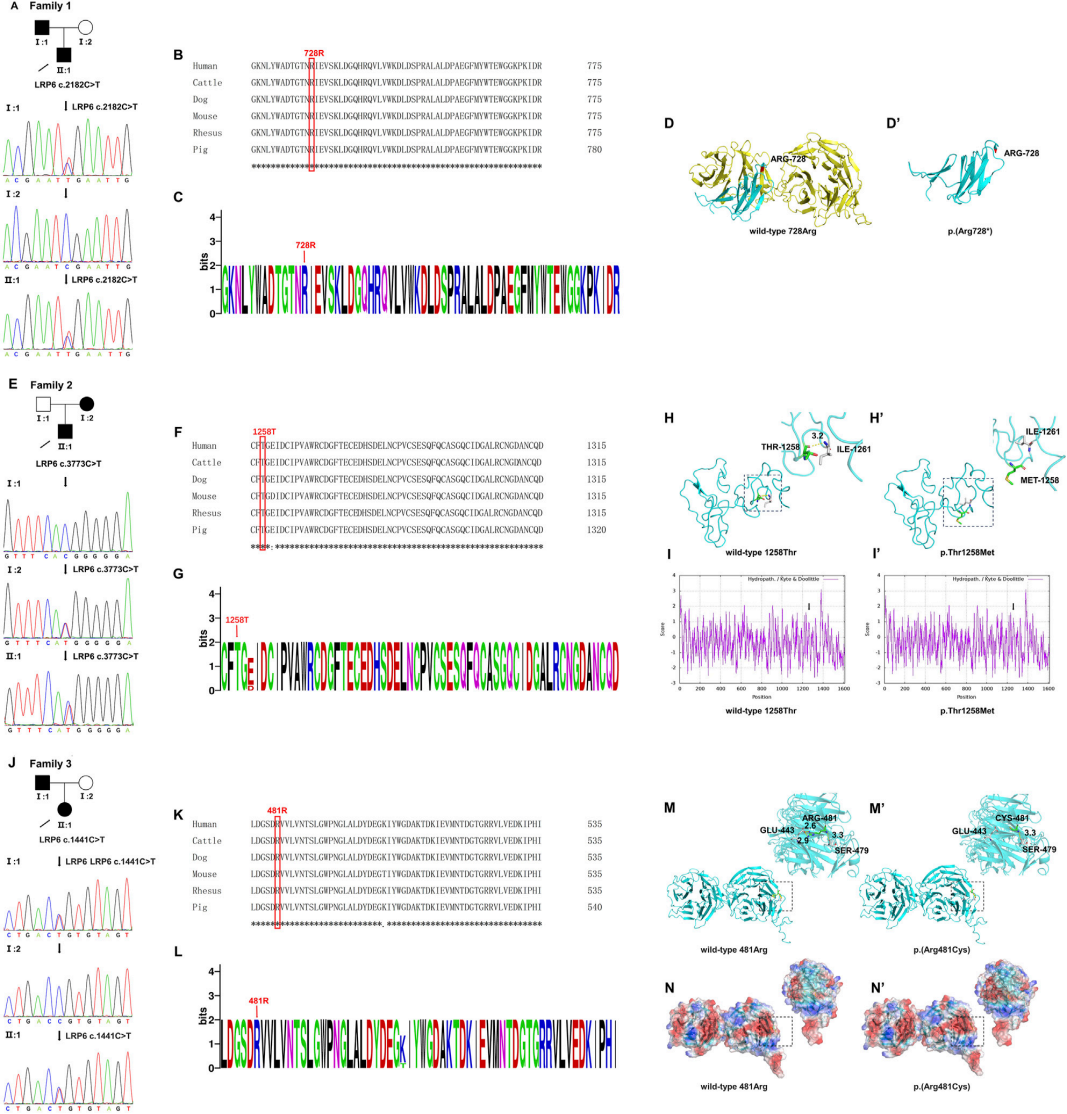
Conserved sequence analysis and structural modeling of three novel LRP6 variants. **(A)** A new *LRP6* truncation variant [c.2182C>T (p.Arg728*)] identified in proband II:1 and her father (I:1) in family 1. **(B,C)** Arg728 is highly conserved across all six species. **(D)** Structural modeling of wild-type Arg728 and **(D′)** p. Arg728*. **(E)** A new *LRP6* missense variant [c.3773C>T (p.Thr1258Met)] identified in proband II:1 and her mother (I:2) in family 2. **(F–G)** Thr1258 is highly conserved across all six species. **(H)** Structural modeling of wild-type Thr1258 and **(H′)** p. Thr1258Met. **(I)** Hydrophobicity analysis of wild-type Thr1258 and **(I′)** p. Thr1258Met. **(J)** A new *LRP6* missense variant [c.1441C>T (p.Arg481Cys)] identified in proband II:1 and her father **(I)** 1 in family 3. **(K–L)** Arg481 was highly conserved across all six species. **(M)** Structural modeling of wild-type Arg481 and **(M′)** p. Arg481Cys. **(N)** Electrostatic potential surface of wild-type Arg481 and **(N′)** p. Arg481Cys. The mutant allele is marked in a red box. * indicates a completely conserved column of amino acids.

Whole-exome sequencing of 35 core families led to the identification of three novel variants, predicted to be pathogenic by SIFT, PROVEAN, PolyPhen-2, and Mutation Taster (Supplementary Table S1), per American College of Medical Genetics classification guidelines. These variants—c.2182C>T (p.Arg728*) (Figure 2A), c.3773C>T (p.Thr1258Met) (Figure 2E), and c.1441C>T (p.Arg481Cys) (Figure 2J)—were confirmed by Sanger sequencing and co-segregation, and showed autosomal dominant inheritance patterns. Arg728* and Arg481Cys were located in YWTD-β-propeller-EGF domains YWTD-propeller domains-EGFs, and Thr1258Met was located in the LDLR type A repeat domain (Figure 4A). The evolutionary conservation of these variants was demonstrated through WebLogo analysis, which showed high conservation of arginine at positions 481 and 728 and of threonine at position 1,258 (Figures 2B,C,F,G,K,L).

We further examined spatial structure changes caused by these variants. The Arg728* variant (cytosine to adenosine at base 2,182) introduced a premature stop codon at position 728, truncating the protein and causing significant structural alterations (Figures 2D,D’). The Thr1258Met variant (cytosine to adenosine at base 3,773) replaced hydrophilic threonine at position 1,258 with hydrophobic methionine, increasing residue hydrophobicity (Figures 2I,I’) and disrupting a hydrogen bond (3.2 Å) between threonine 1,258 and isoleucine 1,261, thus compromising protein stability (Figures 2H,H’). The Arg481Cys variant (cytosine to adenosine at base 1,441) replaced positively charged arginine with uncharged cysteine in position 481, altering the local charge distribution and reducing amino acid potential (Figures 2N,N’). In addition, three-dimensional modeling revealed that arginine 481 formed three hydrogen bonds with glutamic acid 443 and serine 479, stabilizing the protein structure. However, the Arg481Cys variant eliminated the hydrogen bond between cysteine 481 and glutamate 443, leaving only a single bond with serine 479 at a length of 3.3 Å, ultimately affecting LRP6 stability (Figures 2M,M’).

### 3.3 *LRP6* variants impaired β-catenin expression and attenuated *TCF*/*LEF* transcriptional activity

**FIGURE 3:**
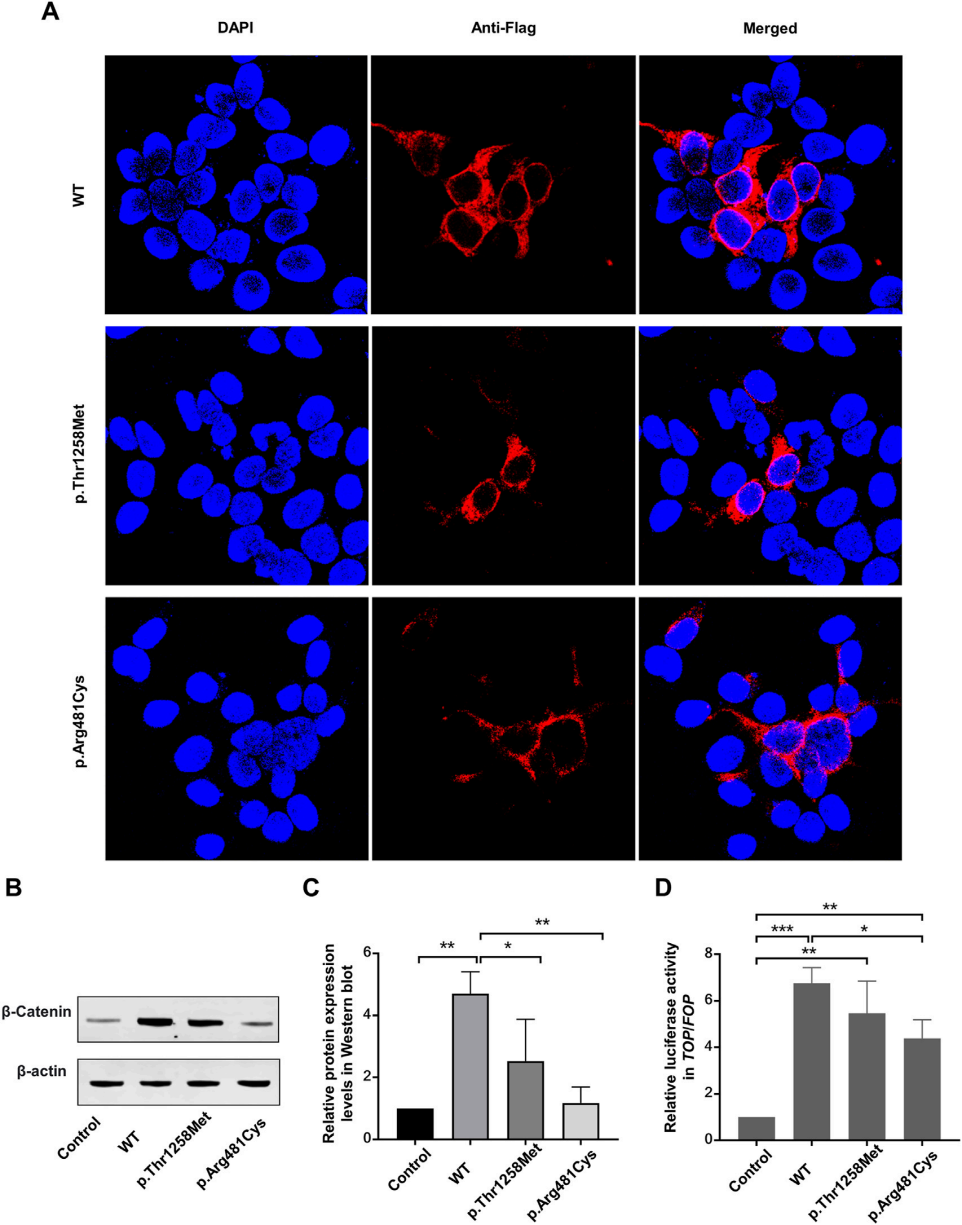
*In vitro* data. **(A)** Subcellular localization of LRP6 before and after variant. **(B,C)** β-catenin expression in LRP6 mutants, determined by Western blotting. Quantitative data are presented. **(D)** Activity of the WNT signaling pathway in HEK-293T cells, measured using TOP-Flash/FOP-Flash luciferase reporter assay. *P < 0.05, **P < 0.01, ***P < 0.001.

Subcellular localization analysis indicated that wild-type LRP6 and the Thr1258Met and Arg481Cys variants localized to the cytoplasm and cell membrane, suggesting that these variants did not affect LRP6 localization (Figure 3A). Western blot analysis revealed significantly elevated β-catenin expression in the wild-type group compared to both the control group and all variant groups (p < 0.05). No statistically significant differences were detected between the control group and any variants (Figures 3B,C). Subsequent TOP/FOP-Flash luciferase reporter assays confirmed that both wild-type LRP6 and all variants retained the ability to significantly activate *TCF*/*LEF* transcriptional activity compared to control. Notably, all variants displayed attenuated *TCF*/*LEF* activation capacity compared to the wild-type group, with the Arg481Val variant showing statistically significant reduction in transcriptional activity relative to wild-type LRP6 (p < 0.05) (Figure 3D).

### 3.4 Association between *LRP6* variants and NSO

**FIGURE 4:**
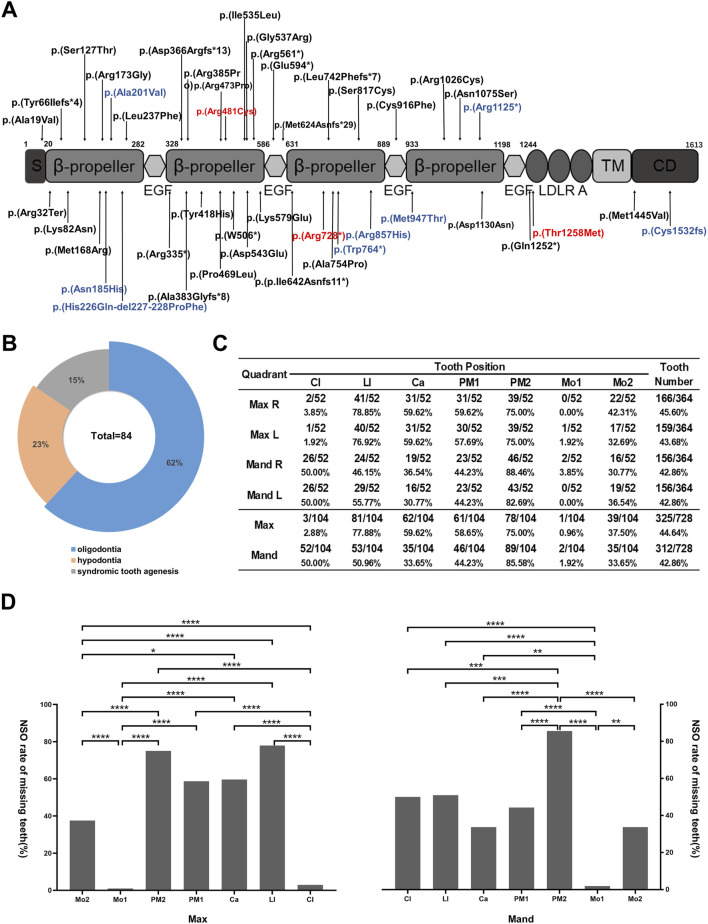
Pattern of *LRP6*-related non-syndromic tooth agenesis. **(A)** Schematic diagram of wild-type LRP6, indicating the distribution of all *L*RP6 variants in patients with tooth agenesis. Red arrows indicate variants identified in this study, and blue arrows indicate *LRP6*-related syndromic phenotype variant sites. **(B)** Proportions of *LRP6* variant types. **(C)** Analysis of missing teeth in 52 NSO patients with *LRP6* variants, showing tooth loss at each permanent dentition position (excluding third molars). **(D)** Tooth loss rate in NSO patients with *LRP6* variants (excluding third molars). Mo, molar; PM, premolar; Ca, canine; LI, lateral incisor; CI, central incisor; Max, maxillary; Mand, mandibular. *P < 0.05, **P < 0.01, ***P < 0.001, ****P < 0.0001.

We reviewed 84 cases of congenital tooth loss associated with 51 *LRP6* variants, including 3 *LRP6* variants reported in this study and previously published data (Supplementary Table S2). Among these 84 patients, 52 patients (62%) presented with *LRP6*-related NSO, 19 patients (23%) with non-syndromic hypodontia, and 13 patients (15%) with syndromic tooth agenesis (Figure 4B). Furthermore, 39 unique *LRP6* variants were identified in the 52 patients with NSO, highlighting a significant association between *LRP6* variants and NSO (Supplementary Table S3).

### 3.5 Genotype-phenotype analysis in NSO patients

Despite numerous *LRP6*-related genotype–phenotype analyses of *LRP6*-related conditions, the specific pattern of *LRP6*-associated NSO remains poorly understood. We found that teeth most frequently missing in *LRP6*-associated NSO were the lower second premolars (85.58%), upper lateral incisors (77.88%), and upper second premolars (75.00%), excluding third molars. Conversely, teeth least likely to be missing were the upper first molars (0.96%), lower first molars (1.92%), and upper central incisors (2.88%) (Figure 4C). The statistically significant differences in missing rates across tooth positions are depicted in Figure 4D (P < 0.05). Interestingly, the missing rate of the maxillary incisors was 2.88% and that of the mandibular incisors was 50%. Moreover, the missing rates of the maxillary lateral incisors (77.88%) and mandibular lateral incisors (50.96%) sharply contrasted with those of the central incisors (Figure 4C). The NSO phenotype exhibited symmetry between the left and right sides but notable asymmetry between the maxilla and mandible (Figure 4C).

## 4 Discussion

LRP6 is an essential co-receptor in the WNT/β-catenin signaling pathway, expressed in both dental epithelial and mesenchymal cells (Yu et al., 2021). During mouse tooth development, LRP6 expression undergoes dynamic changes (Yu et al., 2021), being confined to epithelial cells between embryonic day 11.5 (E11.5) and E13.5, and expanding to both the dental epithelium and papilla by E14.5. These findings suggest that LRP6 plays an important role in tooth morphogenesis. We summarized 48 *LRP6* variants linked to congenital tooth agenesis in humans, and identified a novel truncating variant (c.2182C>T, p. Arg728*) and two novel missense variants [c.3773C>T (p.Thr1258Met) and c.1441C>T (p.Arg481Cys)]. Our results provide a scientific basis for precise genetic counseling and expanding the spectrum of congenital tooth agenesis.

Kantaputra et al., (2022) first associated *LRP6* variants with mesiodens, fused teeth, odontomas, cone-shaped teeth, unseparated roots, and taurodontism. In this study, all three families exhibited similar clinical characteristics (Figures 1A–C). Patients with *LRP6*-associated congenital tooth agenesis most frequently lacked upper lateral incisors and upper and lower second premolars, while upper central incisors and first and second molars were relatively unaffected, aligning with findings reported by Chu et al., 2021; Chu et al., 2021; Zhang et al., 2021; Zhang et al., 2021). We further characterized the phenotype of *LRP6*-related non-syndromic hypodontia, confirming that the maxillary lateral incisor, mandibular second premolar, and maxillary second premolar were the most commonly missing teeth, consistent with *LRP6*-related NSO trends (Supplementary Figure S1). However, the *LRP6*-related syndromic phenotype exhibited different patterns, with the mandibular first molar, maxillary central incisor, and mandibular second molar being the least frequently missing, while mandibular lateral and central incisors were more commonly absent. These findings suggest that *LRP6*-related NSO is more likely to affect the second premolars, while the syndromic phenotype more frequently impacts the mandibular incisors (Supplementary Figure S1). The preferential binding of WNT proteins and their inhibitors to distinct YWTD-β-propeller-EGF domain regions on LRP6 may partially explain the significant heterogeneity in missing tooth patterns associated with LRP6 variants (Chu et al., 2021). Consistent with previously reported data (Cheng et al., 2011), we observed that patients with congenital tooth loss and high bone density syndrome, caused by LRP6 variants, predominantly exhibited variants concentrated in the LRP6-E1 β-propeller. This region is closely associated with the binding of DKK1 and SOST to the LRP6-E1 β-propeller of LRP5/6. Variants in the binding site of LRP5/6 lead to a selective loss of affinity for DKK1 and SOST, resulting in failure to inhibit the WNT signaling pathway and the development of high bone density syndrome (Bourhis et al., 2011). However, this evidence alone does not fully explain the phenotypic heterogeneity, which warrants further investigation.

The activation of the WNT/β-catenin signaling pathway requires WNT ligand-induced aggregation and dimerization/oligomerization of LRP6, a process mediated by the LDLR domain (Bilic et al., 2007; Chen et al., 2014; Cong et al., 2004). Several missense variants in the LDLR domain have been identified in patients with familial hypercholesteremia, resulting in the attenuation of WNT/β-catenin signaling activation (Vijayakumar et al., 2017). To date, only one variant (p.Gln1252*) in the LDLR domain has been implicated in congenital tooth agenesis, but further functional validation has not been conducted (Chu et al., 2021). Herein we identified a second LRP6 variant [c.3773C>T (p.Thr1258Met)] located in the LDLR domain. This variant disrupted a hydrogen bond and increased the hydrophobicity of localized amino acid side-chain motifs, altering the spatial 3D conformation of the protein. This conformational change may impair downstream activation of the WNT/β-catenin signaling pathway mediated by the LDLR domain. We confirmed, through TOP-Flash/FOP-Flash luciferase reporter assay and Western blotting, that the Thr1258Met variant attenuated WNT/β-catenin signaling activity, although the variant retained partial activation capacity in HEK-293T cells.

In addition, we identified the p. Arg728* truncation variant in the LRP6-E3 β-propeller. This variant introduced a premature termination codon, resulting in a truncated LRP6 protein lacking certain extracellular domains, as well as all transmembrane and intracellular domains. Previous studies have shown that LRP6 variants lacking the cytoplasmic domain act as dominant-negative receptors that inhibit the activation of the WNT/β-catenin signaling pathway (Tamai et al., 2000). *In vitro* studies further demonstrate that truncated LRP6 proteins inhibit canonical WNT signaling and cause oligodontia (Yu et al., 2021).

The LRP6-E2 β-propeller consists of P1E1P2E2 functional units and is the primary binding site for WNT1, WNT2, WNT2b, WNT6, and WNT9b (Tsutsumi et al., 2023). In this study, we identified a novel p. Arg481Cys missense variant in the LRP6-E2 β-propeller. This variant was observed to reduce the local amino acid potential and disrupt hydrogen bonding within LRP6, altering its conformation and impairing its function as a receptor in the WNT/β-catenin signaling pathway. Our findings confirmed that the Arg481Cys variant significantly reduced the activation of the WNT/β-catenin signaling pathway. The c.1441C>T (p.Arg481Cys) variant closely resembles the previously identified c.1514A>G (p.Tyr505Cys) variant, also located in the LRP6-E2 β-propeller, which has been shown to markedly reduce WNT signaling activation (Shi et al., 2018).

LRP6 exhibits canonical plasma membrane localization under physiological conditions, serving as a critical co-receptor for WNT/β-catenin signaling activation. This spatial organization requires intact secretory trafficking through the ER-Golgi apparatus, with its signal peptide directing cotranslational translocation to the membrane compartment (Owji et al., 2018). Pathogenic variants disrupting the signal peptide cleavage site (e.g., p. Ala19Val) induce ER retention by impairing signal peptide processing, as mechanistically demonstrated by Massink et al. (2015). In this study, both the p. Thr1258Met missense variant in the LDLR type A repeat domain and the p. Arg481Cys missense variant in the LRP6-E2 β-propeller did not alter the subcellular localization of LRP6, as confirmed by immunofluorescence.

To conclude, we identified three novel *LRP6* variants in patients with NSO from three Chinese families. Our findings provide genetic and functional evidence linking *LRP6* variants to non-syndromic tooth agenesis and expand the variant spectrum for congenital tooth agenesis. Furthermore, we for the first time demonstrate that a missense variant in the LDLR domain attenuates the activation of the WNT signaling pathway *in vitro*. Finally, we summarized the genotype–phenotype correlation for *LRP6*-related NSO, finding that *LRP6* variants are most likely to affect the mandibular second premolars, while maxillary first molars are the least likely to be affected.

## Data Availability

The data presented in this study are deposited in the Genome Sequence Archive (Genomics, Proteomics & Bioinformatics 2021) in National Genomics Data Center (Nucleic Acids Res 2024), China National Center for Bioinformation / Beijing Institute of Genomics, Chinese Academy of Sciences (GSA-Human: HRA011672). These data are publicly accessible at https://ngdc.cncb.ac.cn/gsa-human.
