# Supplementary material for: Genotype–phenotype analysis and functional study of three novel LRP6 variants in non-syndromic oligodontia
Source: Front Genet. 2025 Jun 4;16:1598907. doi: 10.3389/fgene.2025.1598907 (PMC12174413; doi:10.3389/fgene.2025.1598907)
Supplement: Supplementary file 4 [file Image1.pdf]

Supplementary Material

a

Quadrant	Tooth Position							Tooth Number
	CI	LI	Ca	PM1	PM2	Mo1	Mo2	
Max R	2/84<br>2.38%	60/84<br>71.43%	40/84<br>47.62%	38/84<br>45.24%	45/84<br>53.57%	1/84<br>1.19%	25/84<br>29.76%	211/588<br>35.88%
Max L	1/84<br>1.19%	59/84<br>70.24%	40/84<br>47.62%	33/84<br>39.29%	48/84<br>57.14%	2/84<br>2.38%	19/84<br>22.62%	202/588<br>34.35%
Mand R	32/84<br>38.10%	31/84<br>36.90%	22/84<br>26.19%	26/84<br>30.95%	57/84<br>67.86%	2/84<br>2.38%	16/84<br>19.05%	186/588<br>31.63%
Mand L	33/84<br>39.29%	36/84<br>42.86%	19/84<br>22.62%	28/84<br>33.33%	52/84<br>61.90%	0/84<br>0.00%	20/84<br>23.81%	188/588<br>31.97%
Max	3/168<br>1.79%	119/168<br>70.83%	80/168<br>47.62%	71/168<br>42.26%	93/168<br>55.36%	3/168<br>1.79%	44/168<br>26.19%	413/1176<br>35.12%
Mand	65/168<br>38.69%	67/168<br>39.88%	41/168<br>24.40%	54/168<br>32.14%	109/168<br>64.88%	2/168<br>1.19%	36/168<br>21.43%	374/1176<br>31.80%

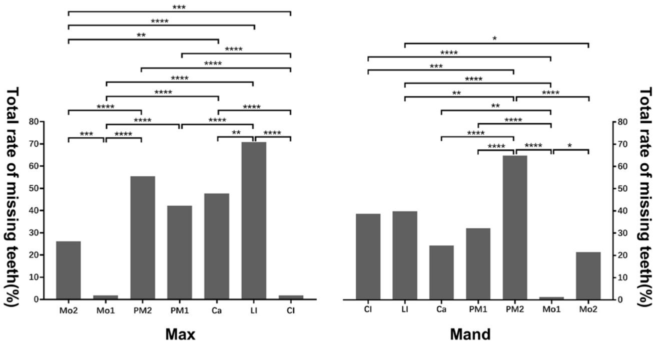

b

Quadrant	Hypodontia Tooth Position							Tooth Number
	CI	LI	Ca	PM1	PM2	Mo1	Mo2	
Max R	0/19<br>0.00%	7/19<br>36.84%	4/19<br>21.05%	3/19<br>15.79%	2/19<br>10.53%	0/19<br>0.00%	1/19<br>5.26%	17/133<br>12.78%
Max L	0/19<br>0.00%	8/19<br>42.11%	3/19<br>15.79%	0/19<br>0.00%	5/19<br>26.32%	0/19<br>0.00%	0/19<br>0.00%	16/133<br>12.03%
Mand R	0/19<br>0.00%	0/19<br>0.00%	0/19<br>0.00%	0/19<br>0.00%	7/19<br>36.84%	0/19<br>0.00%	0/19<br>0.00%	7/133<br>5.26%
Mand L	1/19<br>5.26%	0/19<br>0.00%	0/19<br>0.00%	3/19<br>15.79%	5/19<br>26.32%	0/19<br>0.00%	0/19<br>0.00%	9/133<br>6.77%
Max	0/38<br>0.00%	15/38<br>39.47%	7/38<br>18.42%	3/38<br>7.89%	7/38<br>18.42%	0/38<br>0.00%	1/38<br>2.63%	33/266<br>12.41%
Mand	1/38<br>2.63%	0/38<br>0.00%	0/38<br>0.00%	3/38<br>7.89%	12/38<br>31.58%	0/38<br>0.00%	0/38<br>0.00%	16/266<br>6.02%

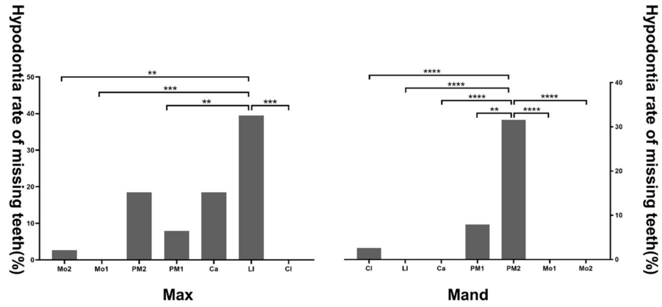

c

Quadrant	Syndromic Tooth Agenesis Position							Tooth Number
	CI	LI	Ca	PM1	PM2	Mo1	Mo2	
Max R	0/13<br>0.00%	12/13<br>92.31%	5/13<br>38.46%	4/13<br>30.77%	4/13<br>30.77%	1/13<br>7.69%	2/13<br>15.38%	28/91<br>30.77%
Max L	0/13<br>0.00%	11/13<br>84.62%	6/13<br>46.15%	3/13<br>23.08%	4/13<br>30.77%	1/13<br>7.69%	2/13<br>15.38%	27/91<br>29.67%
Mand R	6/13<br>46.15%	7/13<br>53.85%	3/13<br>23.08%	3/13<br>23.08%	4/13<br>30.77%	0/13<br>0.00%	0/13<br>0.00%	23/91<br>25.27%
Mand L	6/13<br>46.15%	7/13<br>53.85%	3/13<br>23.08%	2/13<br>15.38%	4/13<br>30.77%	0/13<br>0.00%	1/13<br>7.69%	23/91<br>25.27%
Max	0/26<br>0.00%	23/26<br>88.46%	11/26<br>42.31%	7/26<br>26.92%	8/26<br>30.77%	2/26<br>7.69%	4/26<br>15.38%	55/182<br>30.22%
Mand	12/26<br>46.15%	14/26<br>53.85%	6/26<br>23.08%	5/26<br>19.23%	8/26<br>30.77%	0/26<br>0.00%	1/26<br>3.85%	46/182<br>25.27%

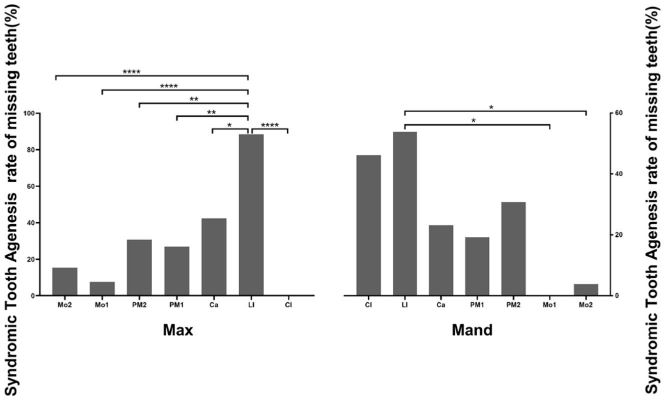

Supplementary Figure S1

Patterns of congenital tooth loss associated with LRP6. (a) Tooth missing rate of all types tooth agenesis patients with LRP6 mutations (excluding the third molars). (b) Tooth missing rate of hypodontia patients with LRP6 mutations (excluding the third molars). (c) Tooth missing rate of syndromic tooth agenesis patients with LRP6 mutations (excluding the third molars)
